# Corneal epithelial ingrowth after perforating corneal injury: a case report

**DOI:** 10.1186/s12886-022-02670-x

**Published:** 2022-12-23

**Authors:** Adrien Quintin, Loïc Hamon, Fidelis A. Flockerzi, Ursula Schlötzer-Schrehardt, Matthias Dias Blak, Berthold Seitz

**Affiliations:** 1 Department of Ophthalmology, Saarland University Medical Centre (UKS), Homburg/Saar, Germany; 2 Department of Pathology, Saarland University Medical Centre (UKS), Homburg/Saar, Germany; 3 Department of Ophthalmology, University of Erlangen-Nürnberg, Erlangen, Germany; 4 Department of Ophthalmology, Klinikum Stuttgart, Stuttgart, Germany

**Keywords:** Corneal perforation, Epithelial invasion, Oak processionary moth caterpillar hair, Laser-assisted blepharoplasty, Case report

## Abstract

**Background:**

Epithelial ingrowth is a rare complication after ocular perforation and can become manifest many years after the primary trauma.

**Case presentation:**

A 49-year-old patient presented with a positive Seidel test of unclear origin at her left eye, as well as a sharply defined anterior-stromal corneal scar at both eyes. Prior operations included a bilateral laser-assisted blepharoplasty 3 months earlier. The patient indicated to have been on holiday to France 5 months earlier, during an ongoing oak processionary moth caterpillars infestation.

The examination using confocal microscopy confirmed a corneal perforation at the left eye and revealed corneal epithelial ingrowth capped with scarred stroma in both eyes. We performed a penetrating keratoplasty at the left eye. The scarred and perforated host cornea was divided into 4 pieces for further investigation: microbiology (negative), virology (negative), histology and transmission electron microscopy (TEM). Histology revealed differently structured epithelium, centrally inverted into the stroma through defects in Bowman’s layer. TEM revealed full thickness corneal perforation with an epithelial plug extending to the lower third of the cornea, but without evidence of epithelial cell migration into the anterior chamber.

Our differential diagnosis of the unclear positive Seidel test with epithelial ingrowth was as follows: (1) corneal perforation by hairs of the oak processionary moth caterpillar, although no hairs could be found histologically; (2) corneal perforation during laser-assisted blepharoplasty, which may be supported by the presence of pigmented cells on the posterior surface of Descemet´s membrane, pointing to a possible iris injury.

**Conclusion:**

Consequently, we highlighted that contact lenses can be useful, safe and inexpensive protective devices in upper eyelid procedures to protect the cornea against mechanical iatrogenic trauma.

## Background

Epithelial ingrowth is a rare complication after ocular perforation and can become manifest many years after the primary trauma [1, 2]. This complication was also described after corneal surgery such as refractive procedures. Indeed, the incidence of epithelial ingrowth after LASIK, graded after the Probst/Machat classification [3], was reported to be 0.03–9.1%, depending on whether the flap was guided with a femtosecond laser or a mechanical microkeratome.[4] Previous histological examination of eyes with epithelial ingrowth after open globe injury revealed a diffuse epithelial invasion in 12%, and a cystic epithelial downgrowth in 88% of the cases [1]. Transition of cystic into diffuse epithelial ingrowth should be avoided, as the possible subsequent secondary glaucoma may be resistant to therapy and lead to blindness. Therefore, in the event of cystic epithelial invasion, laser or surgical opening of the cyst is not recommended. [1] On the contrary, block excision in toto with tectonic penetrating keratoplasty is considered the therapy of choice by some microsurgeon in such cases [1], providing that the excision (and thus the involvement of the cystic extension) does not exceed 150°—or five clock hours—of the circumference of the ciliary body region, in order to prevent postsurgical ocular hypotony. [5]

## Case presentation

A 49-year-old female patient was referred because of new corneal scars of unclear origin in both eyes, after presenting with reduction in visual acuity (left worse than right), first noticed 6 months earlier. The best-corrected decimal visual acuity was 0.6 (-2.50/-0.75/1°) at the right eye and 0.4 (-2.75/-1.75/4°) at the left eye. The patient, who had undergone a strabism operation 45 years earlier, revealed that she had indeed never seen optimally out of her left eye (amblyopia e strabismo). Other prior operations included a bilateral blepharoplasty 3 months earlier, as well as a threefold injectable dermal fillers procedure in the previous year.

**Fig. 1:**
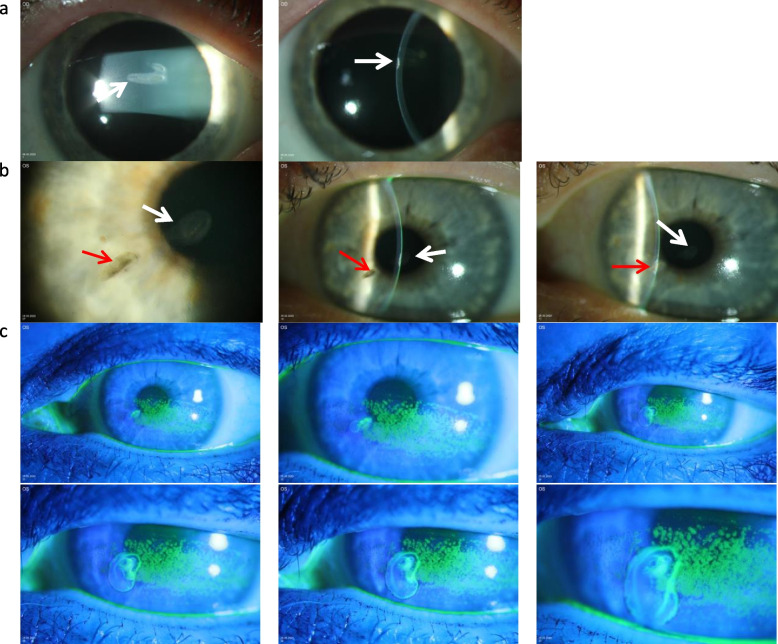
Slit lamp examination at initial presentation. **a** Right eye. Paracentral sharply defined anterior-stromal corneal scar (white arrow). **b** Left eye. Paracentral sharply defined anterior-stromal corneal scar (white arrow) and midperipheral pigmented lesion (red arrow) running through the whole stroma. **c** Positive Seidel test, left eye. After Fluorescein staining, inferiorly emphasized keratitis supericialis punctata as well as clearly positive Seidel test of the midperipheral corneal finding

The intraocular pressure by applanation tonometry was 12 mmHg and 10 mmHg at the right and left eye, respectively. Examinations at the slit lamp revealed a dry eye syndrome as well as a sharply defined anterior-stromal corneal scar at both eyes, located paracentrally at the right (Fig. 1a) and left eye (Fig. 1b). In addition, the left eye showed a midperipheral pigmented lesion with small cyst-like inclusions and small pigmented specky endothelial “precipitates” (Fig. 1b). This alteration ran through the whole stroma and showed a positive Seidel test using blue light after staining with fluorescein (Fig. 1c). This fluorescein staining cannot be triggered purely by a dry eye syndrome. The anterior chamber depth was regular and equal at both sides, and no cells could be identified in the anterior chamber. No iris transillumination could be detected and both lenses were clear.

Funduscopic examination as well as optical coherence tomography of the macula showed a regular finding on both sides. The corneal sensitivity was subjectively equal on both sides, with a significantly higher motion-triggering sensitivity at a thread length of 40 mm at the right eye and 45 mm at the left eye, measured by means of the Cochet-Bonnet aesthesiometer (Luneau Technology, Pont de l’Arche, France). Using a conversion chart provided by the aesthesiometer manufacturer, this corresponds to a pressure of respectively 9.5 mg/S at the right eye and 8 mg/S at the left eye (S = 0.0113 mm^2^ sectional area of the filament), or of respectively 0.8 g/mm [2] and 0.7 g/mm^2^.

The patient stated having no history of trauma or accident with a foreign body, and did neither take any oral nor ophthalmological medication. Following a more precise anamnesis, the patient indicated that she had been on holiday to France 5 months earlier, during a known ongoing oak processionary moth caterpillars infestation, and to ride mountain bike, climb and practice yoga. The patient was referred with a bandage contact lens as well as an initial topical therapy with Moxifloxacin eye drops q.i.d. (4 × /day) for avoiding a bacterial superinfection.

**Fig. 2:**
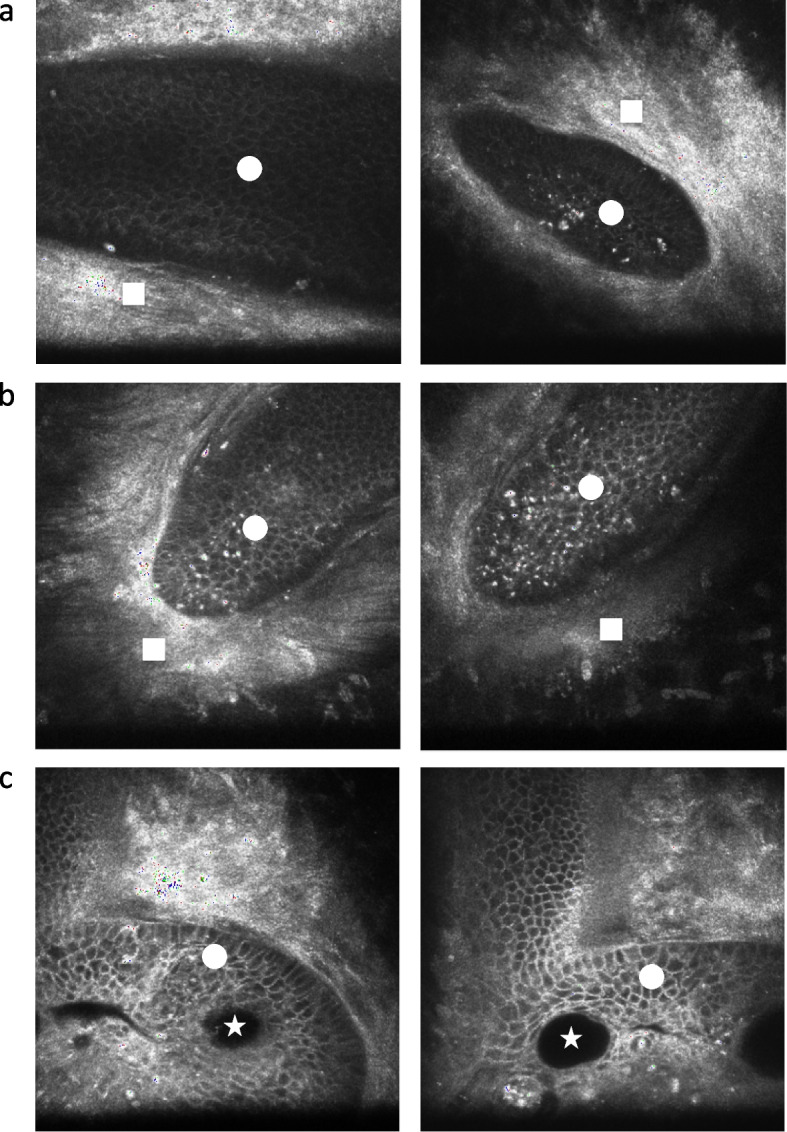
Confocal microscopy at initial presentation a. Right eye. Corneal epithelial ingrowth (white circle) with scarred cap (white square) (focus of depth: 170 μm, 393 μm) b. Left eye. Corneal epithelial ingrowth (white circle) with scarred cap (white square) (focus of depth: 120 μm, 265 μm) c. Left eye. Corneal perforation (white star) surrounded with epithelial cells (white circle) (focus of depth: 249 μm)

The examination using in vivo confocal microscopy (Heidelberg, Retina Tomograph III with Rostock corneal module, Heidelberg Engineering GmbH, Dossenheim, Germany) revealed a corneal epithelial ingrowth capped with a scarred structure in both eyes (Fig. 2a, b) and confirmed the corneal perforation at the left eye (Fig. 2c). The examination using the anterior segment optical coherence tomograph (AS‑OCT) CASIA 2 (Tomey Corp., Nagoya, Japan) also revealed a scar-covered corneal lesion at both eyes, reaching the anterior-stroma at the right eye but running through the whole cornea at the left eye.

**Fig. 3:**
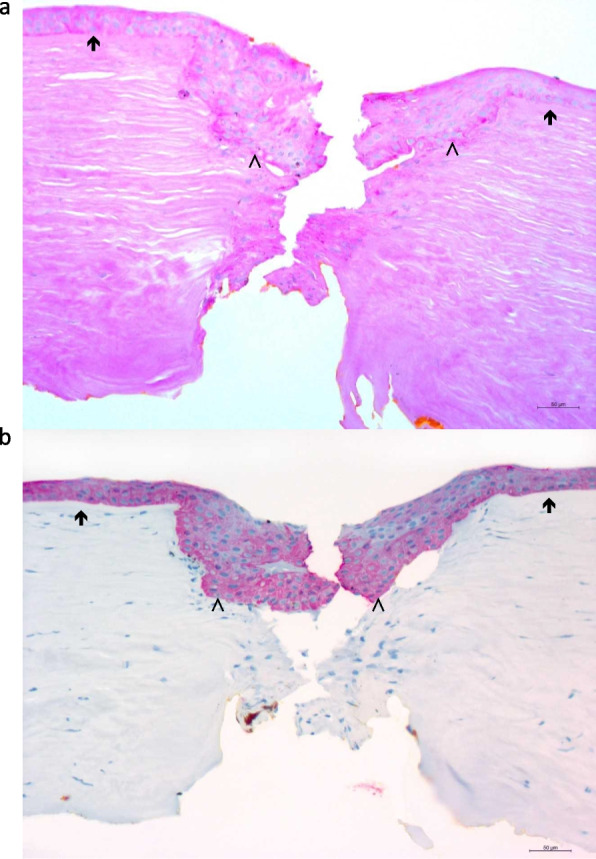
Cross-sectional histologic specimen of the cornea (left eye). **a**/**b** High power view of the cornea showing a centrally disrupted Bowman's layer (^) with initial infiltration of the epithelium into the stroma (*). The Bowman's layer is intact (↑) on the margins. (**a**) PAS staining, (**b**) immunohistochemical staining for pancytokeratin (AE1/3). Image resolution: 2584 × 1936

Our differential diagnosis of the unclear positive Seidel test was as follows:

- corneal perforation during laser-assisted blepharoplasty.
- corneal perforation by foreign body granuloma (hairs of the oak processionary moth caterpillar).
- corneal perforation during filler surgery.

We performed a penetrating keratoplasty at the left eye using a Barron trephine. The diameter of the graft was 8.25 mm, sutured in a patient opening of 8.0 mm diameter with the double continuous cross stitch technique according to Hoffmann. Preoperatively, the corneal button was tomographically measured in a sterile manner in order to minimize refractive surprises after keratoplasty [6–8]. The surgery was uneventful and no surgical complications occured. The scarred and perforated host cornea was divided into 4 pieces for further investigation: microbiology, virology, histology and transmission electron microscopy (TEM). Additionally, aqueous humour was removed intraoperatively for further virological examination (Polymerase chain reaction—[PCR]). Postoperative therapy involved topical corticosteroids (prednisolone acetate eye drops 5 × /day, tapered by 1 drop every 6 weeks), Ofloxacin eye drops (5 × /day for 2 weeks) as well as artificial tears (5 × /day). One year after an uncomplicated course following keratoplasty, the corneal graft appeared clear without sign of rejection. No signs of intraocular irritation could be detected.

### Microbiology of the host cornea

Both the bacterial and fungal polymerase chain reaction (PCR) [9] of the corneal abrasion were negative. The culture showed no growth neither after 7 days, nor after 4 weeks.

### Virology

Neither Epstein-Barr virus (EBV)-, Cytomegalovirus (CMV)-, Herpes simplex virus (HSV)- nor Varicella zoster virus (VZV)-specific DNA was detectable in the PCR of both the corneal biopsy and the anterior chamber aspirate. Consequently, there was no indication of a florid infection.

### Histology

Histological examination (periodic acid-Schiff, Alcian blue, hematoxylin and eosin, Masson–Goldner and Congo red stain) of the host cornea revealed an irregular epithelium, which became centrally ingrown into a fissure of the corneal stroma through defects in Bowman´s layer (Fig. 3a, b). Descemet´s membrane appeared intact and the endothelial cells attached. Neither congophilic nor mucoid deposits could be detected. (Microscopes: Zeiss Axioskop 40; objective lenses: Zeiss A-Plan × 10/0,45, Zoom 6,3 × TV2/3""C; camera: AxioCam MRc5, software: ZEN 3.2 (ZEN lite); Pixel: 2584 × 1936.)

**Fig. 4:**
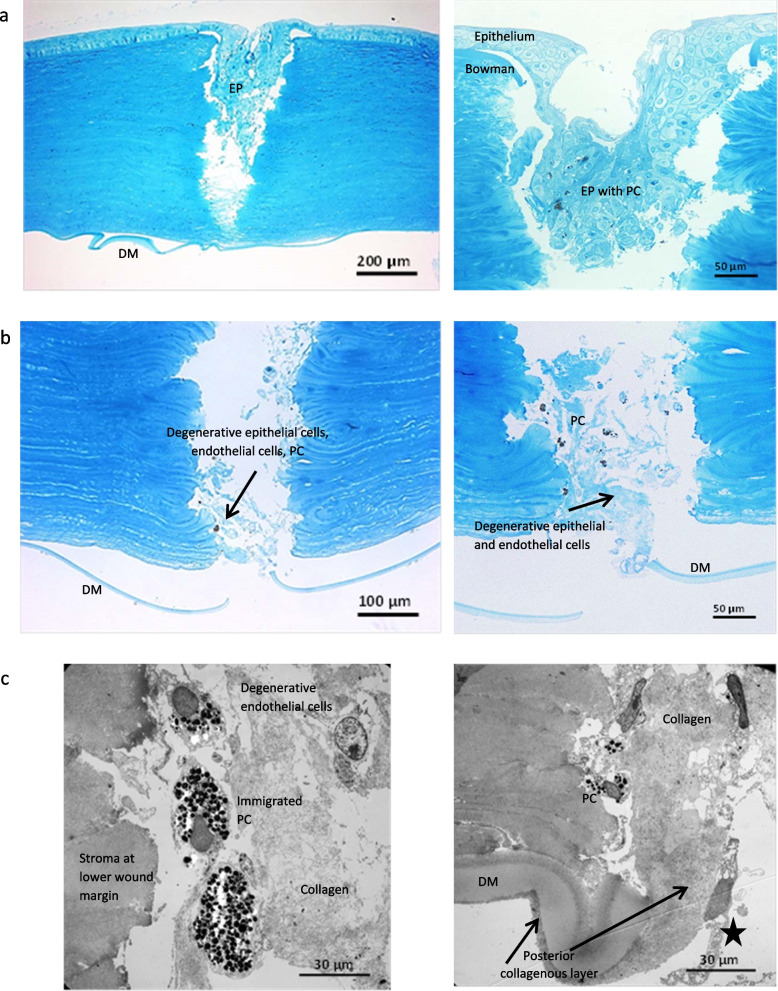
Light and transmission electron microscopy of the host cornea (left eye) **a**. Corneal perforation with epithelial plug (anterior 2/3 of the cornea) containing pigmented cells. EP = epithelial plug. DM = Descemet´s membrane. PC = pigmented cells. Measured resolution of images is not known. Detectors are not known, filters were not used. **b**. degenerative epithelial cell remains (black arrow) and strongly pigmented cells resembling pigmented cells from the irisstroma (small melanin granules) and iris pigment epithelium (large melanin granules) in the lower wound gap (posterior 1/3 of the cornea). PC = pigmented cells. DM = Descemet´s membrane. Measured resolution of images is not known. Detectors are not known, filters were not used. **c**. Extension into the wound gap of the posterior collagenous layer (arrow) on the ruptured Descemet´s membrane, and immigrating endothelial cells (star). PC = pigmented cells. DM = Descemet´s membrane. Measured resolution of images is not known

One micron-thick semithin section (toluidine blue) showed a full thickness perforation of the cornea with an epithelial plug extending to the lower third of the corneal stroma and degenerative epithelial cell remnants interspersed with pigmented cells underneath (Fig. 4a). Pigmented cells could be focally also seen within the epithelial plug (Fig. 4a) and adherent to the posterior surface of Descemet’s membrane (not shown). Despite rupture of Descemet’s membrane, there was no evidence of epithelial cell migration into the anterior chamber (Fig. 4b). (Light microscope: Olympus BX51 [Olympus, Hamburg, Germany], ColorView camera [Soft Imaging Systems, Münster, Germany], Cell^F software [Olympus]).

### Transmission electron microscopy

Transmission electron micrographs (electron microscope EM 906E, Carl Zeiss Microscopy, Oberkochen, Germany) confirmed the presence of degenerative epithelial cells and pigmented cells, resembling iris stroma or iris epithelial cells, within the posterior wound (Fig. 4c). The posterior collagenous layer of Descemet’s membrane was found to extend into the wound gap from the posterior surface, along with remnants of degenerated endothelial cells. No signs of moth caterpillar hairs or other foreign bodies could be detected in serial sections. (Transmission electron microscope: LEO 906E [Carl Zeiss Microscopy, Oberkochen, Germany], integrated plate camera [Carl Zeiss Microscopy], Analysis software package [Soft Imaging Systems]).

## Discussion and conclusion

Corneal trauma with or without foreign body can be poorly symptomatic or even asymptomatic for a long period. Complications such as infections, development of granuloma or epithelial ingrowth can occur and lead the patient to consult. Epithelial ingrowth is a rare complication after corneal injury or surgery.[1, 2] In the case presented above, we performed a penetrating keratoplasty in regards to the associated corneal perforation.

Hereby, we elaborate the above-mentioned differential diagnosis of the unclear positive Seidel test with epithelial ingrowth:

- 1. In 2019, an outbreak of ***oak processionary moth caterpillar*** (Thaumetopoea processionea) has been detected across multiple sites in Europe. The larvae, which hatch around the beginning of May, live in clusters in dense nests on oak trees. It is assumed that their massive reproduction is due to favourable climatic conditions (high temperatures and low rainfall in late spring) during larval development. [10, 11]. Before turning into moths, those moth caterpillars are covered with over half a million tiny stinging hairs (setae) with a length of around 100–500 µm and a diameter of 3–7 µm, [12] which are easily disseminated with the wind due to their small size and weight. The exposure to these hairs have been associated with a range of health effects of varying severity.

The damage caused by moth caterpillar hairs has a double origin: both mechanical, through their ability to migrate into tissues, and allergic, through the release of deleterious toxins and histamine by the protein thaumetopoein, which degranulates mast cells [12]. Those damages may occur months after exposure [13]. The majority of health problems caused by the oak processionary moth caterpillar hair is of dermatologic nature. Indeed, exposure can typically cause a contact dermatitis with burning, itching and redness, but also uncommonly a life-threatening anaphylactic reaction, as described in rare cases. [12]

Few ocular involvements, grouped under the entity of Ophtalmia nodosa, such as eyelid swelling, conjunctivitis and keratoconjunctivitis have been reported. Although they are of rare occurrence, affections of deeper ocular structures such as uveitis (anterior, intermediate and posterior), or even less commonly chorioretinitis and papillitis, have also been described.[13] In a retrospective study from India, moth caterpillar hairs led to penetration of intraocular structures in 3.5% of patients, whereby the time until infiltration of intraocular structures ranged from a few days to 6 months. [14] The presence of intracorneal hairs has previously been identified as a significant risk factor for intraocular penetration, wherefore a consequent surgical removal of the hairs has been recommended. [14]

The hypothesis of the oak processionary moth caterpillar hair being the causal factor of the corneal perforation presented above is theoretically (geographically and temporally) plausible. Hence, the importance of wearing protective glasses bicycling and playing or working outdoors during caterpillar season should be emphasised. Nevertheless, in our case, no moth caterpillar hairs or other foreign bodies could be found neither histologically nor with transmission electron microscopy.

- **2. Soft tissue fillers** are a mainstay in today's minimally invasive facial rejuvenation procedures because of their rapid results and minimal recovery period. A possible periocular indication for this procedure is a tear trough deformity, which is a natural consequence of the anatomic attachments of the periorbital tissues and is characterised by a sunken appearance of the eye that results in a dark shadow over the lower eyelid. This gives the patient a fatigued appearance despite sufficient rest, and is generally refractory to attempts of cosmetic camouflage. [15]. Although associated with a low complication rate, soft tissue fillers are not without any risk. Possible complications range from mild superficial skin irregularities to vascular occlusion leading to skin necrosis or even blindness due to central retinal artery occlusion. [16]. To our knowledge, corneal perforation has not been described yet as a possible complication of soft tissue fillers procedure, which makes this causal hypothesis in the presented case very unlikely.
- **3.** Blepharoplasty is one of the most frequently performed oculoplastic procedures. For the past years, ***laser-assisted blepharoplasty*** has been performed accompanied by several advantages but unfortunately also some complications. Indeed, corneal perforation is one possible serious complication after laser-assisted blepharoplasty, which has been described by some authors. [17, 18]. The hypothesis of an iatrogenic laser beam being the causal factor of the corneal perforation presented above is – to our opinion – the most probable due to the presence of pigmented cells (small melanin granules resembling pigmented cells from the iris stroma and large melanin granules resembling pigmented cells from the iridal pigment epithelium) on the back of Descemet´s membrane, shedding light on a possible iris injury. Contact lenses have been suggested as useful, safe and inexpensive protective devices in upper eyelid procedures to shelter the cornea against mechanical iatrogenic trauma. A previous study recommended for example the use of a metallic scleral contact lens as a protection during laser procedures. [19]. Despite this protective measure, corneal perforation could still develop because of a Bell's phenomenon with elevation of the cornea superior to the corneal shield. [17]. Therefore, meticulous awareness of the laser power and exposure on periocular tissue must always be kept in mind during laser-assisted blepharoplasty to avoid undesirable intraocular side effects and postoperative outcomes. [18]

## Data Availability

The data that support the findings of this study are available from the Department of Ophthalmology, Saarland University Medical Center (UKS) (Homburg/Saar, Germany) but restrictions apply to the availability of these data, which are not publicly available. Data are however available from the authors upon reasonable request and with permission of the patient.

## Abbreviations

CMV: Cytomegalovirus
EBV: Epstein-Barr virus
HSV: Herpes simplex virus
PCR: Polymerase chain reaction
TEM: Transmission electron microscopy
VZV: Varicella zoster virus
